# Wharton's Jelly Derived Mesenchymal Stem Cells: Future of Regenerative Medicine? Recent Findings and Clinical Significance

**DOI:** 10.1155/2015/430847

**Published:** 2015-03-15

**Authors:** Ilona Kalaszczynska, Katarzyna Ferdyn

**Affiliations:** ^1^Department of Histology and Embryology, Center for Biostructure Research, Medical University of Warsaw, Chalubinskiego 5, 02-004 Warsaw, Poland; ^2^Centre for Preclinical Research and Technology, Banacha 1b, 02-097 Warsaw, Poland; ^3^Polish Stem Cell Bank, Grzybowska 2/41, 00-131 Warsaw, Poland

## Abstract

Around 5 million annual births in EU and 131 million worldwide give a unique opportunity to collect lifesaving Wharton's jelly derived mesenchymal stem cells (WJ-MSC). Evidences that these cells possess therapeutic properties are constantly accumulating. Collection of WJ-MSC is done at the time of delivery and it is easy and devoid of side effects associated with collection of adult stem cells from bone marrow or adipose tissue. Likewise, their rate of proliferation, immune privileged status, lack of ethical concerns, nontumorigenic properties make them ideal for both autologous and allogeneic use in regenerative medicine applications. This review provides an outline of the recent findings related to WJ-MSC therapeutic effects and possible advantage they possess over MSC from other sources. Results of first clinical trials conducted to treat immune disorders are highlighted.

## 1. Introduction

Interest in mesenchymal stem cells has been kindled in 1960s as the result of Friedenstein's observations who reported that the bone marrow stroma can generate bone [1]. It was later shown that bone marrow stromal cells have chondrogenic and adipogenic properties and a high ability for self-renewal [2]. Even though there is debate on the technical name (mesenchymal or multipotent stem cells), there is an agreement to the acronym “MSC”. Since their original description, presence of MSC has been proven in many adult and embryonic tissues such as adipose tissue [3], muscle [4], peripheral blood [5], lung [6], heart [7], corneal stroma [8], dental pulp [9], placenta [10], endometrium [11], amniotic membrane [12], and Wharton's jelly [13]. MSC have the capability to differentiate into wide range of specialized cells of mesodermal origin: bone cells, cartilage, fat, cardiomyocytes, muscle fibers, renal tubular cells, and break germ layer commitment and differentiate into cells of ectodermal origin, for example, neurons, and endodermal origin, such as hepatocytes and pancreatic islets cells. Due to the above properties, MSC are considered as a new emerging treatment option and therapeutic agent in regenerative medicine. MSC therapeutic potential can be executed by direct replacement of injured tissue cells or by paracrine effect on surrounding environment, indirectly supporting revascularisation, protecting tissue from stress-induced apoptosis, and appropriately modulating inflammatory reaction. Results of MSC-based cell therapies are very promising in various clinical fields, based on* in vitro* and* in vivo *research results and more than 400 clinical trials registered.

## 2. Are All MSC Phenotypically and Functionally Equivalent? Age Does Matter

The lifelong perseverance of adult MSC in the body makes them particularly susceptible to the accumulation of cellular damage, which can lead to cell death, senescence, or loss of regenerative function and in extreme cases to neoplastic transformation. In contrast, neonatal MSC such as Wharton's jelly derived MSC, in their short, prenatal life are spared from proaging factors. Decreased repair capacity and increased susceptibility to degenerative diseases may stem from the fact that the function of stem cells declines with age. There is increasing evidence that the age of the donor tissue affects several properties of mesenchymal stem cells [14–16]. By means of single cell transcriptional analysis, it was shown that aged adipose tissue derived MSC (ADSC) are significantly compromised in their ability to support the vascular network formation and are unable to rescue age-associated impairments in cutaneous wound healing [17]. Further, bone marrow derived MSC have lesser myogenic potential and engraftment properties than developmentally early MSC [18]. As recently shown, one of the mechanisms implicated in MSC aging involves Akt/mTOR pathway and its inhibition prevents the development of age-related phenotype and maintains MSC morphology, self-renewal, and differentiation capacity [19]. Further studies demonstrate that the expression levels of inflammatory response genes change with age and that the age-dependent decrease in expression of several cytokine and chemokine receptors is important for the migration and activation of BMSC. By adoptive transfer of aged BMSC to young endotoxemic mice, authors showed that aged cells lacked the anti-inflammatory, protective effect of their young counterparts, which indicate that BMSC undergo an age-related decline in their immunomodulatory activity [20].

A growing body of evidence suggests that elevated activity of certain proteins can have beneficial effects on aging and aging-related diseases. Among them is SITR1, NAD+-dependent protein deacetylase, which is downregulated in rodent bone marrow derived MSC with aging [21] or human MSC with increasing passages [22]. It is also shown that expression of genes related to senescence such as CHEK1, p16^ink4a^ increases in ADSC with age where at the same time proapoptotic regulators levels, ATR, TNF*α*, and NF*κ*B decreased [23]. Aging alters the availability of CD45^−^/CD34^+^/CD133^+^ ADSC and their angiogenic properties [24].

Healthspan of mesenchymal stem cells also depends on maintaining physiological level of reactive oxygen species (ROS). However, during lifetime and exposure to environmental stress, ROS levels can increase dramatically. This may result in significant damage to cell structures and promote MSC aging. In support of the hypotheses, increased levels of ROS have been reported in aging BMSC [14]. Furthermore, exposure of adult ADSC isolated from old rat donors to H_2_O_2_ resulted in decreased expression of integrin and reduced phosphorylation of focal adhesion kinase Src and FAK. In consequence, intramyocardial transplantation of aged ADSC into acute myocardial infarction model rats resulted in a decreased survival rate of old MSC in the infarct region. The authors conclude that the old ADSC are more sensitive to the microenvironmental ROS and their therapeutic effectiveness is impaired [25]. In another study, in a rat myocardial infarction model, authors evaluated regenerative capacity of human MSC derived from young versus older patients (1–5 versus 50–70 years old). “Young” MSC outperformed “older” MSC in cardiac parameters: ejection fraction, fractional shortening, and left ventricular end-diastolic and end-systolic volumes. Increase in vascular density and decrease in metalloproteinases levels and activity were observed in recipients of “young” BMSC [26]. Similarly, MSC obtained from young individuals have been induced to undergo neuroectodermal differentiation* in vitro*, but this effect could not be reproduced in BMSC from elderly individuals [27]. This proves adult BMSC unsuitable for successful cell replacement strategies for neurologic diseases in elderly patients in autologous setting.

During normal aging cells divide and telomeres that are essential to maintain the stability of genomes shorten. Even though MSC in their niche are relatively quiescent, adult MSC during their lifespan undergo significantly more divisions shortening their telomeres than neonatal cells. Thus, in comparison to MSC from adult tissues, WJ-MSC at such an early embryonic state retain telomere at highest possible length, which protects them from premature loss of viability.

A very important issue, which does not apply to Wharton's jelly derived MSC, is an exposure of adult MSC during the lifetime to intrinsic (e.g., inflammatory mediators) and extrinsic factors, for example, nonsteroidal anti-inflammatory drugs (NSAIDs) commonly used in patients to treat inflammation, pain, and fever. These factors may greatly inflect MSC viability or plasticity. Effects of NSAIDs on the MSC potential for proliferation and differentiation towards the osteogenic and chondrogenic lineages were investigated [28]. It was shown that type X collagen, a marker of late stage chondrocyte hypertrophy, is constitutively expressed by mesenchymal stem cells (MSC) from osteoarthritis patients treated with NSAID, Naproxen [29]. Similarly, osteogenic differentiation of MSC was affected, and downregulation of mineral deposition in the extracellular matrix was observed [30]. These results contradicted previous findings, demonstrating no effect of several types of NSAIDs on osteogenic differentiation. However,* in vitro* chondrogenesis, shown by glycosaminoglycans production, was significantly inhibited. These findings suggest that NSAIDs may inhibit MSC chondrogenic differentiation and disrupt endochondral bone formation [31]. Despite discrepancies, it is evident that NSAID can alter certain essential processes involved in the MSC performance as therapeutic agent.

The therapeutic potential of adult MSC can be also affected by donors lifestyle. Although high-fat diet induced type 2 diabetes did not affect the number of cells per gram of adipose tissue, analysis of differentiation potential of ADSC derived from high-fat diets fed mice showed a higher adipogenic potential and a lower endothelial differentiation potential* in vitro *compared to control group [32]. Impaired response to osteogenic stimuli was also shown for ADSC from obese patients.* Ranx2* expression was 6–9 times lower than in control cells and mineralization nodules were fewer and smaller [33]. Altered properties of ADSC and BMSC were also demonstrated by others. Surprisingly, in obese mice, increased frequency of BMSC and subcutaneous ADSC was shown. However, adipogenic, osteogenic, and chondrogenic potential of BMSC from obese mice was diminished. ADSC showed increased adipogenic and osteogenic differentiation but decreased CD105 expression consistent with inefficient chondrogenic potential [34]. Observed phenotype might be associated with increased levels of free fatty acids (FFA) in plasma of obese patients. Consistent with this notion, palmitate (most abundant FFA in plasma of obese patients) treated BMSC showed induced expressions of adipogenic transcription factors, namely, CCAAT enhancer-binding protein, C/EBP*β*, C/EBP*α*, and PPAR*γ*, and in consequence increased adipogenic differentiation [35]. The elevated level of FFA in obese individuals may initiate events leading to irreversible changes in MSC from bone marrow and adipose tissue. Consistently, another study confirmed upregulation of adipocyte lineage commitment genes, such as* Tcf 21*,* Pitx2*, and* Lif*. At the same time, the expression of “stemness” genes (*Sdf1*,* Tbx15*) was downregulated [36].

Obesity is one of the factors increasing the risk of developing type 2 diabetes [37]. Metabolic diseases such as diabetes may influence stem cell niche and endogenous MSC properties. Therefore feasibility of autologous stem cell therapy in diabetic patients may not be possible or at least significantly hampered. Indeed, it was shown that BMSC from diabetic patients, although phenotypically similar to healthy human BMSC, expressed insulin, C-peptide, and other pancreatic markers not observed in control healthy cells [38]. Furthermore, in a recent study, investigators demonstrated that diabetes alters ADSC milieu and diminishes the cells' ability to establish a vascular network both* in vitro* and* in vivo* in wound healing mouse model [39]. It could be expected, since significant decrease of major angiogenic genes (*Vegf-a, Fgf-2*, and* Pdfg-a*) and their associated receptors (*Cxcr-4*,* Fgfr-2*, and* Pdgfr-a*) expression was observed.

Collectively, this observation indicates that the microenvironment in disease influences the stem cells. Therefore, tissues from patients with various metabolic diseases may not be satisfactory as an autologous source of mesenchymal stem cells for therapeutic purposes [40].

According to WHO statistics, 35% of adults aged 20 and over are overweight and 11% are obese (as of 2008), while 8% are living with diabetes. Taking into account the fact that the passing of time and changes in MSC microenvironment due to disease translate into reduced effectiveness of tissue regeneration, MSC derived from Wharton's jelly offer a good clinical alternative to adult MSC. In the near future, autologous use of these cells will be possible due to growing interest in Wharton's jelly banking.

## 3. More Differences between Adult and Wharton's Jelly Derived MSC

The superiority of WJ-MSC is based not only on adult MSC limitations but on its own prominent capacity.

5 million annual births in EU and 131 million worldwide give a unique opportunity to collect umbilical cord (UC), isolate lifesaving mesenchymal stem cells, and cryopreserve them for allogeneic or autologous application as soon as the need arises. The unlimited availability of tissue source is not the only advantage of WJ-MSC.

### 3.1. Isolation Efficiency: Number Does Matter

Most of clinical applications of MSC require a large number of cells for transplantation. Therefore, abundance, easiness of isolation, and proliferative potential may be deciding factors while choosing a source of MSC. The amount of mesenchymal stem cells, which can be obtained from bone marrow, is very limiting. Only 0.001 to 0.01% of mononuclear cells were reported [41], while 1 g of adipose tissue yields approximately 5 × 10^3^ stem cells, which is 500-fold greater than in the bone marrow [42]. The isolation efficiency from Wharton's jelly is high and ranges from 1 to 5 × 10^4^ cells/cm of umbilical cord [43]. Side-by-side comparison of MSC from bone marrow adipose tissue and Wharton's jelly demonstrated that WJ-MSC have highest proliferative capacity among tested cell types [44]. MSC from the umbilical cord can be isolated either by enzymatic digestion or by explant culture of 1–3 mm pieces of the UC [45–48]. However, at p0 explant culture method yielded 2.8 times more cells per gram of UC than enzymatic digestion [46]. Of great importance for large-scale MSC production is the fact that population doubling time of WJ-MSC isolated by enzymatic method is significantly longer [49]. Furthermore, enzymatic digestion may induce cellular damage, as MSC isolated by explant method demonstrated increased viability. Another advantage of explant method is growth factors release from tissue pieces during* in vitro* culture. Large amounts of different growth factors were reported in Wharton's jelly [46, 50]. Among them, bFGF is noteworthy, as it regulates self-renewal and positively affects osteogenic and chondrogenic differentiation of MSC while added to the growth medium [51–54]. Wharton's jelly released bFGF mediates stimulation of WJ-MSC growth in a way external supplementation provides.

To further increase isolation and culture efficiency, several modifications of explant culture methods and dedicated devices were proposed [55, 56]. Interestingly, a device designed for repeated explant culture at the same time prevented floating of Wharton's jelly pieces [55]. By sequential transfer of device with fragments of tissue strung on the steel rings, investigators reported 15–20 times higher number of cells derived by this method. However, mentioned method seems to be labor-intensive, especially for fast and large-scale production of WJ-MSC. Another approach, proposed by others in order to optimize method of MSC isolation, is based on isolation of WJ-MSC from large pieces or the entire cord piece [56–58]. The only concern posed by this method is possible heterogeneity of derived cells. However, no differences in cell surface antigen expression, population doubling time, or pattern of adipogenic, osteogenic, and chondrogenic differentiation were observed [56]. Therefore, explant culture methods of Wharton's jelly only or entire umbilical cord are worth of consideration for labor-, time-, and cost-effective WJ-MSC isolation for clinical purposes.

### 3.2. Properties of WJ-MSC Crucial for Clinical Application

Phenotypic analysis performed by many groups proved that WJ-MSC fit the minimal criteria outlined for MSC by the International Society for Cellular Therapy [59]. WJ-MSC express mesenchymal markers such as CD73, CD90, and CD105 and are negative for endothelial, CD31, and hematopoietic, CD45, CD34, markers [13, 60, 61]. What sets WJ-MSC apart and makes them more unique and useful for therapeutic applications from adult MSC is their more primitive characteristics [62]. It is already well known that WJ-MSC display several features of embryonic stem cells (ESC), especially regarding the expression of ESC-like stem cell markers and wide spectrum of differentiation beyond mesodermal origin. Expression of pluripotency genes, Oct-4, Nanog, and SOX-2, was reported for WJ-MSC [46, 63, 64], although much lower than in ESC [65]. Modest expression of pluripotency genes might explain why WJ-MSC are not tumorigenic (do not form teratomas) as demonstrated in numerous preclinical studies in immunocompetent and immunodeficient animals [66, 67]. Furthermore, as recently shown by the comprehensive analysis of WJ-MSC and ESC transcriptome, the high expression level of several tumor suppressor genes may explain the lack of* in vivo* teratoma induction [65]. The same mechanism might be one of many responsible for attenuation of tumor growth by WJ-MSC. Moreover, large amounts of various cytokines and growth factors are secreted by WJ-MSC which result in cancer cells* in vitro* and tumor* in vivo* growth inhibition. WJ-MSC cell lysates or conditioned medium inhibited growth of breast adenocarcinoma, ovarian carcinoma, osteosarcoma [68], benign neoplastic keloid cells [69], bladder tumor [70], or lymphoma cells [71]* in vitro*. Similarly, intratumorally administered cell lysates and WJ-MSC conditioned medium inhibited mammary carcinoma, osteosarcoma, and pancreatic and lung tumor growth and resulted in decreased tumor sizes and weights* in vivo* [72–75]. The antitumor effect of WJ-MSC was shown to be accomplished through multiple mechanisms. Antiproliferative properties of WJ-MSC were demonstrated by cell counting, MTT, BrdU or [^3^H]-thymidine incorporation assays, cell cycle regulators, and flow cytometric analysis. In lung or bladder tumor cells, cell cycle progression was blocked in G0/G1 phase and resulted in the downregulation of cyclin A2 and its associated kinase, cdk2, downregulation of Akt, and upregulation of tumor suppressor p53 phosphorylation, as well as cyclin dependent kinases inhibitor, p21 protein level [70, 73, 76]. In the breast cancer cells, synthesis of DNA was inhibited and arrest of cells in G2 phase of the cell cycle observed [74]. By cleaved caspase 3/9 upregulation in cancer cells, WJ-MSC were executing its proapoptotic effect [70, 77]. Consistently, increase in tumor cell death driven by WJ-MSC was due to an inhibitory effect on cancer “survival genes,” such as Bcl-2, Bcl-xL, Survivin, Mcl-1, and cIAP-1 [78]. Autophagy was also indicated as one of the mechanisms responsible for anticancer effect of WJ-MSC. Upregulation of autophagy-related BAX, ATG5, ATG7, and BECLIN-1 genes was observed in osteosarcoma [68] and keloid cells [69] upon treatment with WJ-MSC conditioned medium or lysates.

Still, we must undertake far-reaching precautions and moderate enthusiasm in the implementation of WJ-MSC as anticancer therapy, since reports of tumor supporting function were recently published in regard to esophageal carcinoma [79] and renal cancer [80].

### 3.3. Immunoprivileged Status of WJ-MSC

The ability to modulate immunological responses ranks WJ-MSC as an important compatible stem cell type for therapeutic applications in allogeneic setting. The mechanisms of immunoprivilege are still investigated; however, low MHC-I level and absence of MHC-II expression protect them from NK-mediated lysis [81, 82]. Despite the fact that they synthesize, though low, amounts of MHC class I, WJ-MSC do not demonstrate immunogenicity. It can be attributed to the lack of co-stimulatory molecules-CD 40, CD80, CD86 expression, and high levels of inhibitors of immune response: indoleamine-2,3-dioxygenase (IDO) and prostaglandin E2 (PGE2). Of particular importance is the fact that WJ-MSC express high levels of leukocyte antigen G6 (HLA-G6), the same which is produced by trophoblast and protects the embryo from immune-based destruction [83]. Notably, in such a nonchallenging to allogeneic immune cells setup, immunorejection of WJ-MSC seems not to pose a threat and HLA matching may not be required before MSC transplantation. Therefore, administration of immunosuppressive drugs is not required, thereby protecting the patient against their side effects. Besides mechanisms described above, immunoprivilege of WJ-MSC depends on immunosuppressive functions mediated by the wealth of paracrine factors as well as cell-cell contact (reviewed in detail in Jyothi Prasanna and Jahnavi [84] and Ma et al. [85]).

The question remains if immunoprivilege of allogeneic WJ-MSC upon differentiation is maintained. Although use of allogeneic MSC in clinic is considered safe, reports of limited survival and long-term engraftment of MSC in such a setting are published. For instance, increased immunogenicity of BMCS was shown upon endothelial and myogenic differentiation [86]. A shift in the expression of immune antigens MHC-I and MHC-II made BMSC susceptible to immune rejection in a rat model of myocardial infarction. In another case, when the composite of hydroxyapatite and allogeneic BMSC was implanted, none of the allografts survived or showed osteogenic differentiation. Treatment with FK506 immunosuppressant prevented rejection and stimulated allogeneic BMSC osteogenic differentiation* in vivo* [87]. So far, such discouraging results were not reported for WJ-MSC. Results published so far demonstrated that the chondrogenic differentiation of human WJ-MSC did not change the level of expression of the aforementioned genes except for a very minor increase in the level of MHC class I. Costimulatory factors were not expressed and could not activate T lymphocytes. Moreover, high levels of potent inhibitors of immune response (IDO, HLA-G, and PGE2) were detected in differentiated WJ-MSC [88].* In vivo* analysis of pig WJ-MSC injected into damaged rat brain revealed successful engraftment, proliferation, and differentiation into tyrosine hydroxylase positive neuronal cells without requiring immune suppression [89]. So far, it seems that state of immunoprivilege is stable in WJ-MSC upon multidirectional differentiation. Further studies are required in order to prove sustained immunoprivilege status of WJ-MSC upon differentiation which may depend on the species or stimulating factor.

## 4. Clinical Applications of WJ-MSC

The first clinical trial to test the feasibility and efficacy of WJ-MSC therapy was registered in 2008. By November 2014, the public clinical trials database http://www.clinicaltrials.gov/ has shown 51 clinical trials using WJ-MSC for a very wide range of therapeutic applications (Table 1, keywords used: Wharton's jelly mesenchymal stem cells or umbilical cord mesenchymal stem cells). Most of these trials are safety studies (Phase I) and proof of concept (Phase II) with very few in Phase III (comparison of a new treatment to the standard treatment).

To date, the results of studies listed are not published yet. However, rapidly increasing interest in WJ-MSC clinical application has resulted already in several published observations.

### 4.1. Type 1 Diabetes Mellitus

In a double blind study 15 patients with newly onset type 1 diabetes mellitus received 2 doses of 1.5–3.2 × 10^7^ of WJ-MSC at 4-week interval by intravenous delivery [90]. Strikingly, within a period of 24 months, in 3/15 patients insulin supplementation was discontinued and in 8/15 and 3/15 the daily dosage was reduced by more than 50% and 15–50%, respectively. Only 1 patient did not benefit from WJ-MSC treatment. In the control group, not subjected to WJ-MSC treatment, the dose of insulin increased gradually. No adverse reactions, chronic side effects were reported during the follow-up study.

### 4.2. Type 2 Diabetes Mellitus

In a nonplacebo controlled study, 22 patients (17 on insulin therapy) received WJ-MSC [91]. A first dose of 10^6^/kg was infused intravenously. Five days later, another dose was delivered to the pancreas via the splenic artery. Within 6 months after treatment, from 17 patients receiving insulin, 7 became insulin free and 5 had a reduction in insulin requirement by ≥50%, in the rest ≤50%, with only 1 patient who did not respond to MSC therapy. Interestingly, WJ-MSC treatment resulted in a significant decrease in proinflammatory IL-1*β* and IL-6 plasma level. This may have* in vivo* implications because IL-6 is an osteoclastogenic stimulus. Therefore, treatment of diabetic patients may also protect them from osteoporosis. Such effect may not be achieved by bone marrow derived MSC from aged patients, since BMSC from a mouse model of early aging secrete higher levels of IL-6 and have higher osteoclastogenesis-inducing activity [92]. Moreover, adult aged BMSC cocultured with activated T-cells were found to secrete more IL-6 than younger cells [93].

In both studies, parameters such as levels of glycated hemoglobin, C-peptide, and fasting plasma glucose were monitored. All parameters improved, HbA1c level gradually decreased, and progressive increase of C-peptide and C-peptide/glucose ratio was observed.

### 4.3. Systemic Lupus Erythematosus (SLE)

SLE is common and potentially fatal autoimmune disease resulting in renal, neural, cardiovascular, musculoskeletal, or cutaneous injury. In a nonplacebo controlled study, 40 patients received 2 doses of 10^6^/kg of WJ-MSC at 1-week interval by intravenous delivery [94]. No transplantation related side effects were observed. During 12 months of follow-up study 13/40 and 11/40 achieved major or partial clinical response manifested by significant improvement in renal function, decrease in SLEDAI (Systemic Lupus Erythematosus Disease Activity Index) and BILAG (British Isles Lupus Assessment Group) scoring. 16/40 patients did not respond to MSC therapy. At 9 months after treatment, 7 patients experienced disease relapse; therefore, the authors concluded that repeated infusion with WJ-MSC is necessary to avoid disease relapse.

### 4.4. Late-Onset Hemorrhagic Cystitis (HC)

HC is a common complication after allogeneic hematopoietic stem cell transplantation, characterized by hemorrhagic inflammation of the bladder. Late-onset of HC is frequently associated with ongoing graft-versus-host disease (GVHD). Seven patients received 1–3 doses of 0.8–1.6 × 10^6^/kg WJ-MSC by injection through a central line. As a result of stem cell treatment, gross hematuria dramatically resolved in 2–12 days, while the time to remission for patients not treated with WJ-MSC was significantly longer [95].

Reported results confirm that WJ-MSC are viable option as an adjuvant treatment for late-onset hemorrhagic cystitis.

The above results of pioneering studies demonstrated the effectiveness of WJ-MSC infusion for immune disorders.

## 5. Conclusions

Taken together, the clinical implication of oxidative stress, telomere length, DNA damage and disease is impaired therapeutic potential of MSC isolated from aged patients. These changes in MSC biology indicate that aged patients may require an alternative source of stem cells for treatment. The high efficiency of WJ-MSC recovery, the minimal ethical concerns associated with its acquirement and use, low immunogenicity, and the fact that they are from healthy, young donors make them an ideal source of MSC for autologous and allogeneic applications. Private and public banking of perinatal tissues gains popularity. During MSC preparation for clinical applications, observance of national and international regulations regarding standards and procedures is required. Quality management systems already in place in functioning tissue/cell banks guarantee high standards for the donation, procurement, testing, processing, storage, and distribution of the WJ-MSC. Therefore, as the off-the-shelf product, WJ-MSC can be applied safely, immediately, and on demand. The next several years should abound in results of clinical applications of WJ-MSC and hopefully prove their invaluable properties.

## Figures and Tables

**Table 1:** A summary of clinical trials of WJ-MSC registered on http://www.clinicaltrials.gov/ as of December 2014. ?: not mentioned.

Application	ClinicalTrials.gov identifier	Registered/phase	Number of cells	Regimen	Delivery route	Estimated enrollment
GvHD	NCT01754454	2012/1, 2	10^6^/kg	4× at 1-week intervals	Intravenous	30
	NCT00749164	2008/1, 2	1-2 × 10^6^/kg	?	Intravenous	20

Autism	NCT02192749	2014/1, 2	?	4× at 3-month intervals	Intravenous	20

Multiple sclerosis	NCT02034188	2014/1, 2	?	7× once per day	Intravenous	20
	NCT01364246	2011/1, 2	?	?	?	20

Hereditary cerebellar ataxia	NCT01489267	2011/2	10^7^/2 mL	4× at 3–5-day intervals	Lumbar puncture	20
	NCT01360164	2011/1, 2	?	?	?	20

Amyotrophic lateral sclerosis	NCT01494480	2011/2	?	4× at 3–5-day intervals	Lumbar puncture	30

Hypoxic ischemic encephalopathy	NCT01962233	2013/1	1–8 × 10^8^	single dose	Intravenous	10

Alzheimer's	NCT02054208	2014/1, 2	Low dose: 1 × 10^7^/2 mL High dose: 7.5 × 10^7^/15 mL	3× at 4-week intervals	Intraventricular	40
	NCT01547689	2012/1, 2	0.5 × 10^6^/kg	8× at 2-week interval	Intravenous	30

Spinal cord injury	NCT02237547	2014/1, 2	?	Multiple times over the course of one month	Intravenous and intrathecal	20
	NCT01393977	2011/2	?	?	Lumbar puncture	40
	NCT01873547	2013/3	?	?	Lumbar puncture	300

Cerebral palsy	NCT01929434	2013/3			Lumbar puncture	300

Severe aplastic anemia	NCT02218437	2014/4	0.5–1 × 10^6^/kg	3× at 1-week intervals	?	20
	NCT01182662	2010/2	10^6^/kg	2× at 3-month intervals	Intravenous	30

Myelodysplastic syndromes	NCT01129739	2010/2	10^6^/kg	2× at 3-month intervals	Intravenous	30

Cardiopathy	NCT01739777	2014/1, 2	10^6^/kg	Single dose	Intravenous	30

Dilated cardiomyopathy	NCT01219452	2010/1, 2	?	?	Intramuscular	30

Myocardial infarction	NCT01291329	2011/2	?	?	Intracoronary	160

Autoimmune hepatitis	NCT01661842	2012/1, 2	10^6^/kg	3× at 4-week intervals	Intravenous	100

Lupus nephritis	NCT01539902	2012/2	?	?	Intravenous	25

Systemic lupus erythematosus	NCT01741857	2012/1, 2	?	?	?	40

Epidermolysis bullosa	NCT01033552	2009/2	?	?	Intravenous	75

Burns	NCT01443689	2011/1, 2	?	?	?	20

Diabetic foot ischemia	NCT01216865	2010/1, 2	5 × 10^7^	?	Intramuscular	50

Osteoarthritis	NCT02237846	2014/1, 2	?	3× once daily or single dose	Intravenous or intra-articular	40

Type 1 diabetes	NCT01219465	2010/1, 2	2 × 10^7^	Single dose	Intravenous	50

Type 2 diabetes	NCT01954147	2013/1, 2	?	?	Intravenous	100
	NCT01413035	2011/1, 2	10^6^/kg	2× at 90-day intervals	Intravenous	30

Ulcerative colitis	NCT01221428	2010/1, 2	2 × 10^7^ + 10^7^	One week apart	Intravenous + mesenteric artery	50

Duchenne muscular dystrophy	NCT01610440	2012/1, 2	?	?	?	15
	NCT02235844	2014/1	?	?	?	1

Liver failure	NCT01724398	2012/1, 2	10^5^/kg	4× at 1-week interval	Intravenous	120
	NCT01218464	2010/1, 2	5 × 10^5^/kg	3× at 4-week interval	Intravenous	70
	NCT01844063	2013/1, 2	10^5^, 10^6^, or 10^7^/kg	8× at 1-week intervals	Intravenous	210

Liver cirrhosis	NCT01224327	2010/1, 2	?	Single dose	Via hepatic artery	50
	NCT01233102	2010/1, 2	?	Single dose	Intravenous or via hepatic artery	200
	NCT01220492	2010/1, 2	5 × 10^5^/kg	2× at 4-week intervals	Intravenous	45
	NCT01662973	2012/1, 2	10^6^/kg	3× at 4-week intervals	Intravenous	100
	NCT01877759	2013/1, 2	?	6× at 1-week intervals	Intravenous	20
	NCT01342250	2011/1, 2	?	?	?	20
	NCT01728727	2012/1, 2	10^6^/kg	Single dose	Via hepatic artery	240

Liver transplantation	NCT01690247	2012/1	10^6^/kg	3× at 4-week intervals	Intravenous	50

Ischemic-type biliary lesions	NCT02223897	2014/2, 3	10^6^/kg	4× at 1-week intervals 5× at 4-week intervals	Intravenous	66

HIV infection	NCT01213186	2010/2	Low dose: 5 × 10^5^/kg High dose: 1.5 × 10^6^/kg	At weeks 0, 4, 12, 24, 36, and 48	Intravenous	72

Rheumatoid arthritis	NCT01547091	2012/1, 2	4 × 10^7^	4× at 3-month intervals	Intravenous	200
	NCT01985464	2013/1, 2	?	5× daily	Intravenous	20

Ankylosing spondylitis	NCT01420432	2011/1	10^6^/kg	2× at 3-month intervals	Intravenous	10

Bronchopulmonary dysplasia	NCT01207869	2010/1	3 × 10^6^/kg	Single dose	Via endotracheal tube	10
